# Transoral Robotic Surgery (TORS) for Head and Neck Cancer in the Elderly Population: Functional Outcomes, Survival, and Complications

**DOI:** 10.1002/hed.70097

**Published:** 2025-11-19

**Authors:** Erim Pamuk, Avinash Beharry, Karma Lambercy, Margaux Dalla‐Vale, Nina Wahler, Şefik Hoşal, Christian Simon

**Affiliations:** ^1^ Service d'Oto‐Rhino‐Laryngologie—Chirurgie Cervico‐Faciale, Centre Hospitalier Universitaire Vaudois (CHUV) Université de Lausanne (UNIL) Lausanne Switzerland; ^2^ Department of Otorhinolaryngology, Faculty of Medicine Hacettepe University Ankara Turkey; ^3^ Department of Otorhinolaryngology, Faculty of Medicine Atılım University Ankara Turkey

**Keywords:** age, complications, elderly, functional outcome, head and neck carcinoma, nonelderly, survival, swallowing, TORS, transoral robotic surgery

## Abstract

**Objective:**

To compare functional and oncologic outcomes in elderly (≥ 70 years) and nonelderly (< 70 years) patients after transoral robotic surgery (TORS).

**Methods:**

A retrospective chart review was conducted on 114 patients who underwent TORS for head and neck squamous cell carcinoma between 2012 and 2022. Patient and tumor characteristics, perioperative details, complications, and survival parameters were analyzed. Swallowing function was assessed using the Functional Outcome Swallowing Scale (FOSS).

**Results:**

Of the 114 patients, 37 (32.5%) were elderly, and 77 (67.5%) were nonelderly. Elderly patients had higher comorbidity scores (*p* < 0.001). Oropharyngeal and oral cavity primaries were more common in the nonelderly group, whereas laryngeal primaries predominated in elderly patients (*p* < 0.01). Complication rates were higher in nonelderly (37.6%) than in elderly (18.9%) patients, though not statistically significant (*p* = 0.07). In elderly patients, FOSS scores showed no significant change preoperatively, postoperatively (< 3 months), or at the last follow‐up (median 36 months). The nonelderly group experienced worse early postoperative FOSS scores compared to baseline but showed significant improvement, returning to preoperative levels by the last follow‐up. Nonelderly patients had better FOSS scores at last follow‐up compared to elderly patients (*p* = 0.014). Overall and recurrence‐free survival outcomes were better in the nonelderly group, but disease‐specific survival rates were comparable.

**Conclusion:**

Despite higher comorbidity rates in the elderly, TORS demonstrated favorable complication rates in the elderly population. Swallowing function returned to baseline after 3 months in both groups. TORS appears safe for elderly patients with comparable oncologic outcomes.

## 1 Introduction

Transoral robotic surgery (TORS) has emerged as a minimally invasive approach over recent decades [1, 2]. Since its approval by the Food and Drug Administration (FDA) in 2009, TORS has been employed as an alternative to open surgery for the treatment of head and neck tumors. The majority of TORS indications encompass oropharyngeal cancer, with a more selective application in other head and neck tumors as well as cancer of an unknown primary. The primary advantage of TORS is its capacity to provide excellent visualization of the surgical site without the necessity for external incisions, mandibulotomy or pharyngotomy [3, 4, 5]. This minimally invasive approach has been demonstrated to facilitate superior functional recovery in comparison to conventional open surgical procedures [6].

The treatment of head and neck cancer in the elderly population must be tailored to the individual patient, with due consideration given to the presence of comorbidities and the increased risk of frailty associated with this age group. Consequently, despite the satisfactory functional and survival outcomes of TORS, surgical intervention is often less favored in this demographic compared to younger patients [7]. However, the available data regarding the functional and survival outcomes following TORS in the elderly population are limited and inconclusive. Parhar et al. reported that TORS can provide favorable oncological outcomes and lower perioperative mortality in patients older than 70 with HPV‐driven oropharyngeal cancer [8]. A recent study by Costantino et al. using data from the national cancer database (NCDB), reported similar 30‐day and 90‐day mortality rates between patients younger and older than 70 [9]. Our data will provide long‐term survival and functional outcomes, including perioperative complications, comparing elderly and nonelderly patients following TORS.

## 2 Methods

This study was approved by the Research Ethics Committee of the Canton of Vaud (CER‐VD) (protocol number: 2023‐01258, approval date: 6.6.2023).

### 2.1 Patient Cohort

The medical records of 250 patients who underwent TORS at the Department of Otolaryngology‐Head and Neck Surgery of the University Hospital of Lausanne (CHUV), Lausanne, Switzerland, between July 18, 2012 and December 31, 2022 were retrospectively reviewed. A total of 114 patients who provided written informed consent, had a confirmed pathology report of squamous cell carcinoma (SCC), and were followed up for at least 6 months postoperatively were included in the study.

### 2.2 Study Variables

Data extracted from patient records included demographic characteristics, preoperative disease parameters, operative details, postoperative course, complication status, pathological features, p16 status, functional swallowing status, and survival parameters. The Functional Outcome Swallowing Scale (FOSS) was used to assess patients' swallowing ability before surgery, during the first 3 months postoperatively, and at the median follow‐up of 36 months postoperatively [10], as well as preoperative and intraoperative tracheotomy status and length of hospital stay. FOSS scores were retrospectively extracted from the medical database by an experienced head and neck surgeon. The Charlson Comorbidity Index (CCI) was used to assess comorbidity status and was categorized as mild (≤ 2), moderate (3–4), and severe (≥ 5). The need for and administration of adjuvant treatment was also recorded. Patients were divided into two groups according to age: those aged > 70 years and those aged ≤ 70 years. A cut‐off age of 70 years was used to categorize patients, as this has been used in previous studies [3, 9].

### 2.3 Diagnostic Work‐Up and Surgical Procedure

In accordance with standard institutional protocol, all patients underwent a comprehensive preoperative clinical evaluation and a thorough radiological staging examination. All patients underwent careful triple endoscopy, supplemented by biopsies, and were assessed for suitability for transoral surgery. All treatment decisions were discussed at a weekly multidisciplinary board meeting. The surgical procedure consisted of TORS ± neck dissection performed either during the same surgical session or at a later stage. The Da Vinci system (Intuitive; Sunnyvale, CA) was used for each surgical procedure (SI system from 2012 to 2018 and XI system from 2018 to 2022). A Feyh‐Kastenbauer mouth gag was systematically used for optimal exposure, the 0° and 30° binocular endoscope for 3D vision, and the Maryland forceps and monopolar scissors as instruments. On a case‐by‐case basis, either the dorsal branch of the lingual artery or the superior thyroid artery was clipped transorally if no neck dissection was performed, or the external branch of the carotid artery was ligated transcervically if neck dissection was performed. The resection specimens were oriented by the pathologist using surgical markings or by direct examination with the surgeon. The margins of the resection specimens were inked by the pathologist. Specimens were sectioned approximately every 4 mm and measured either with a ruler if on slide or digitally if scanned. Based on the pathology reports, patients with positive or close margins were offered revision surgery consisting of resection of the affected quadrant with an additional macroscopic margin of 1 cm. Intraoperative frozen sections have also been obtained since 2019 in our institution. Patients with a final positive margin were offered concurrent chemoradiotherapy. Typically, N0 necks underwent selective neck dissection from levels I or II to IV and *N*+ necks underwent selective neck dissection from level I to IV.

### 2.4 Primary and Secondary Outcome Measures

The primary endpoints were overall survival (OS), recurrence‐free survival (RFS), and disease‐specific survival (DSS). OS was defined as the time from diagnosis to death from any cause. RFS was defined as the time from diagnosis to tumor recurrence or death from any cause. DSS was defined as the time from diagnosis to death directly attributable to cancer. Secondary outcome measures were FOSS scores and complication status. In the event that a patient exhibited a missing FOSS score for a specified time period, the patient was excluded from the subsequent analysis, which was conducted with the remaining cases. The complications were classified in accordance with the Clavien–Dindo grading scale [11]. The severity of complications was further categorized as major (grades 3, 4, and 5) and minor (grades 1 and 2) [12]. Additionally, the origins of the complications were classified as either TORS‐related or non‐TORS‐related, as described by Hay et al. [12]. In the event of any instance of bleeding, the Mayo classification was employed for the categorization of the bleeding rate.

### 2.5 Statistical Analysis

Statistical analysis was performed using R (programming language). Survival curves for OS and DSS were plotted using the Kaplan–Meier estimation method and compared using the log‐rank test. Restricted mean survival time comparison was used for RFS as the proportional hazard assumptions were not met. Univariate and multivariate analyses were performed using Cox proportional hazards regression to identify survival and disease‐specific predictors. The Wilcoxon test was used to analyze nonparametric variables in multiple groups. Categorical variables were examined using the *χ* ^2^ test and Fisher's exact test. Student's *t* test was used to compare the means of independent variables. The level of statistical significance was set at *p* < 0.05; all reported *p* values are 2‐sided. ChatGPT‐4o and DeepL were used for grammatical language editing.

## 3 Results

### 3.1 Patient Characteristics

A total of 114 patients with HNSCC who underwent TORS were included in the analysis. Of these patients, 37 (32.5%) were over 70, and 77 (67.5%) were under 70. All patients were followed over a median period of 36 months. The nonelderly group had a median age of 60 (range, 41–69) years, while the elderly group had a median age of 75 (range, 70–85) years. Most of the nonelderly patients were men (75.3%), as were most of the elderly patients (62.1%) (*p* = 0.219). Seven patients (9.1%) in the nonelderly group and five patients (13.5%) in the elderly group had synchronous tumors at the time of diagnosis (*p* = 0.521). There was no significant difference in the rates of concurrent neck dissection between the groups (62% vs. 70%, *p* = 0.414). Sixty‐one patients (79.2%) underwent feeding tube insertion and one patient (1.3%) had a tracheotomy in the nonelderly patient group, whereas 27 patients (73.0%) had feeding tube insertion and one patient (2.7%) underwent a tracheotomy in the elderly patient group (*p* = 0.613 and *p* = 0.546, respectively). Thirty‐nine nonelderly patients (50.7%) had initial surgical margins more than 3 mm, while this was achieved in 10 patients (27.0%) in the elderly group (*p* = 0.015). There was no significant difference between the groups in terms of lymphovascular invasion, perineural invasion and extracapsular spread.(*p* = 0.658, *p* = 0.063 and *p* = 0.174, respectively). Mean CCI scores were higher in the elderly patient group (6.32 ± 1.53 vs. 3.90 ± 1.12, *p* < 0.001). Table 1 presents a comparison of patient and tumor characteristics according to groups.

**TABLE 1:** Patient characteristics according to both groups.

Characteristics	< 70 years old *n* = 77 (%)	≥ 70 years old *n* = 37 (%)	*p*
Charlson Comorbidity Index			**< 0.001**
(Mean ± SD)	3.90 ± 1.12	6.32 ± 1.53	
Tumor site			**< 0.01**
Other	1 (1.3)	3 (8.1)	
Oral cavity	11 (14.3)	1 (2.7)	
Oropharynx	57 (74)	20 (54.1)	
Larynx	3 (3.9)	7 (18.9)	
Unknown primary	5 (6.5)	6 (16.2)	
Stage			0.828
Early [0,1,2]	51 (66.2)	26 (70.3)	
Advanced [3, 4]	26 (33.8)	11 (29.7)	
cT stage			0.344
1	37 (48)	12 (32.4)	
2	22 (28.6)	15 (40.6)	
3–4	2 (2.6)	2 (5.4)	
is‐x	16 (20.8)	8 (21.6)	
cN stage			0.830
0	43 (55.8)	19 (51.4)	
1	15 (19.5)	9 (24.3)	
2–3	19 (24.7)	9 (24.3)	
p16 status			0.772
0	46 (59.7)	20 (54.1)	
1	26 (33.8)	14 (37.8)	
Missing	5 (6.5)	3 (8.1)	
Past primary			1
Yes	26 (33.8)	12 (32.4)	
No	51 (66.2)	25 (67.6)	
Past radiotherapy			1
Yes	13 (16.9)	6 (16.2)	
No	64 (83.1)	31 (83.8)	
Frozen sections			0.917
No	57 (74)	26 (70.3)	
Negative	18 (23.4)	10 (27)	
Positive	2 (2.6)	1 (2.7)	
Post op hospital stay			0.286
1 week	21 (27.3)	9 (24.3)	
2 weeks	31 (40.2)	10 (27)	
3 weeks	13 (16.9)	8 (21.6)	
More than 3 weeks	11 (14.3)	10 (27)	
Postoperative radiotherapy			0.639
Yes	30 (39)	12 (32.4)	
No	47 (61)	25 (67.6)	
Postoperative chemotherapy			0.099^a^
Yes	11 (14.3)	1 (2.7)	
No	66 (85.7)	36 (97.3)	
pT stage			0.810
1	37 (48)	17 (46)	
2	26 (33.8)	11 (29.7)	
3–4	2 (2.6)	2 (5.4)	
Is‐x	12 (15.6)	7 (18.9)	
Revision for margins			0.430
Yes	20 (26)	13 (35.1)	
No	57 (74)	24 (64.9)	
Initial margin status			**0.015**
> 3 mm	39 (50.7)	10 (27)	
1–3 mm	21 (27.3)	20 (54.1)	
< 1 mm	17 (22)	7 (18.9)	

*Note*: Bold indicates statistical significance.

^a^ Close to statistical significance.

### 3.2 Functional Outcomes

**FIGURE 1:**
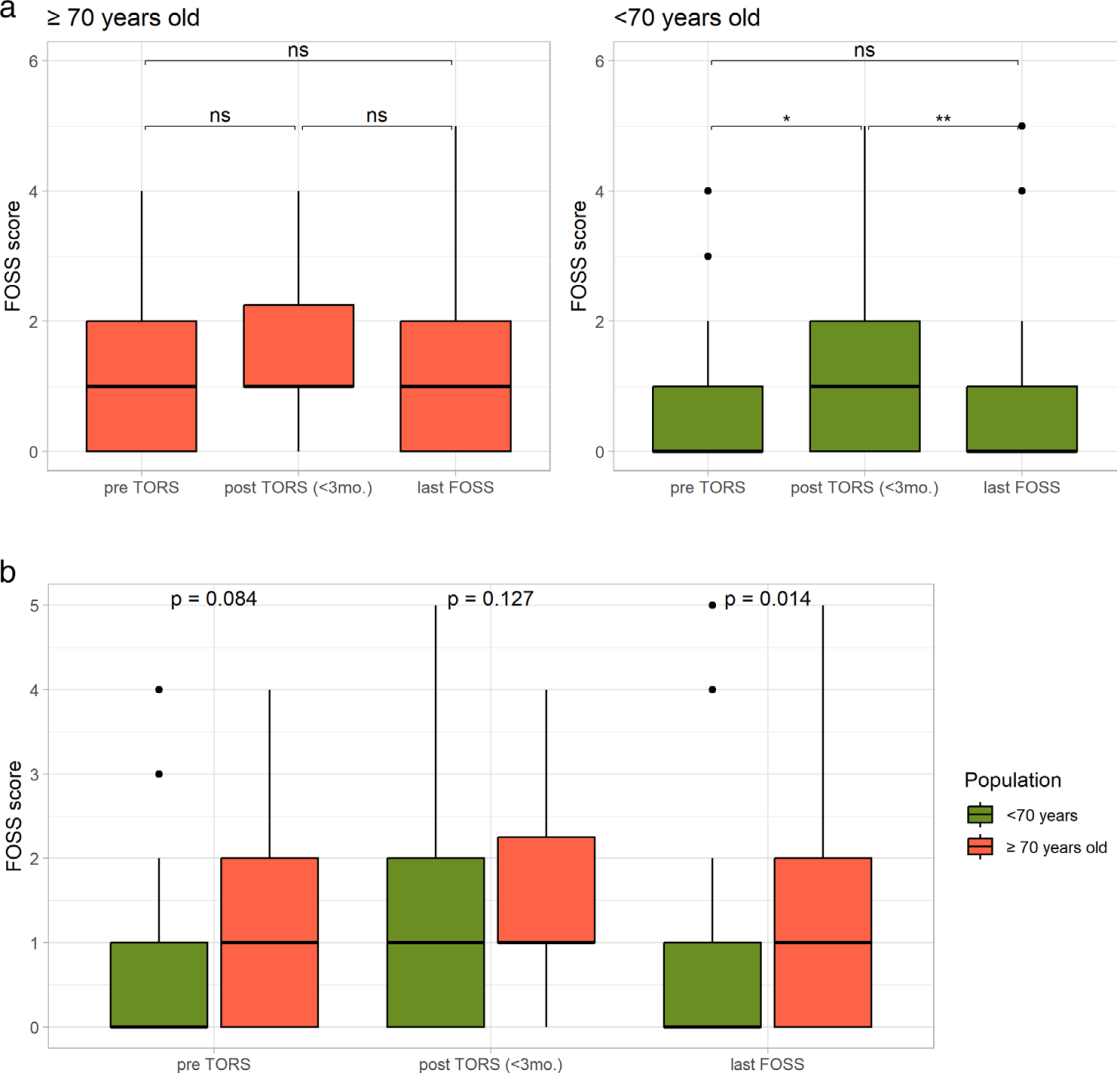
(a) Comparison of FOSS score changes following TORS in elderly and nonelderly patients. (b) Comparison of FOSS scores between the two groups within each surgical time period. *p < 0.05, **p < 0.01. [Color figure can be viewed at wileyonlinelibrary.com]

A graphical representation of the FOSS scores pre‐TORS, post‐TORS (< 3 months), and at the last follow‐up for both elderly and nonelderly groups is presented in Figure 1. There were no significant differences between the pre‐TORS, post‐TORS (< 3 months), and last FOSS scores (median follow‐up of 36 months) of elderly patients. The nonelderly group exhibited significantly worse scores in the early post‐TORS period compared to their preoperative period; however, their last FOSS scores significantly improved and returned to their baseline preoperative levels. The FOSS scores at the last follow‐up were not statistically different from the scores prior to treatment in both groups (Figure 1a). Nevertheless, there was an increase in the number of cases with FOSS scores of four and five at the last follow‐up (Figure S1). Elderly patients had higher FOSS scores at all three time periods compared to nonelderly patients; however, only the last FOSS score difference reached statistical significance (*p* = 0.014) (Figure 1b). There were no significant differences in the rates of low (0‐1‐2) and high (3‐4‐5) FOSS scores between the groups at any time period. Four patients (3.5%) (two in elderly [6.2%] and two in nonelderly [2.9%] group, *p* = 0.445) were completely gastrostomy dependent at the last follow‐up.

### 3.3 Complications

**FIGURE 2:**
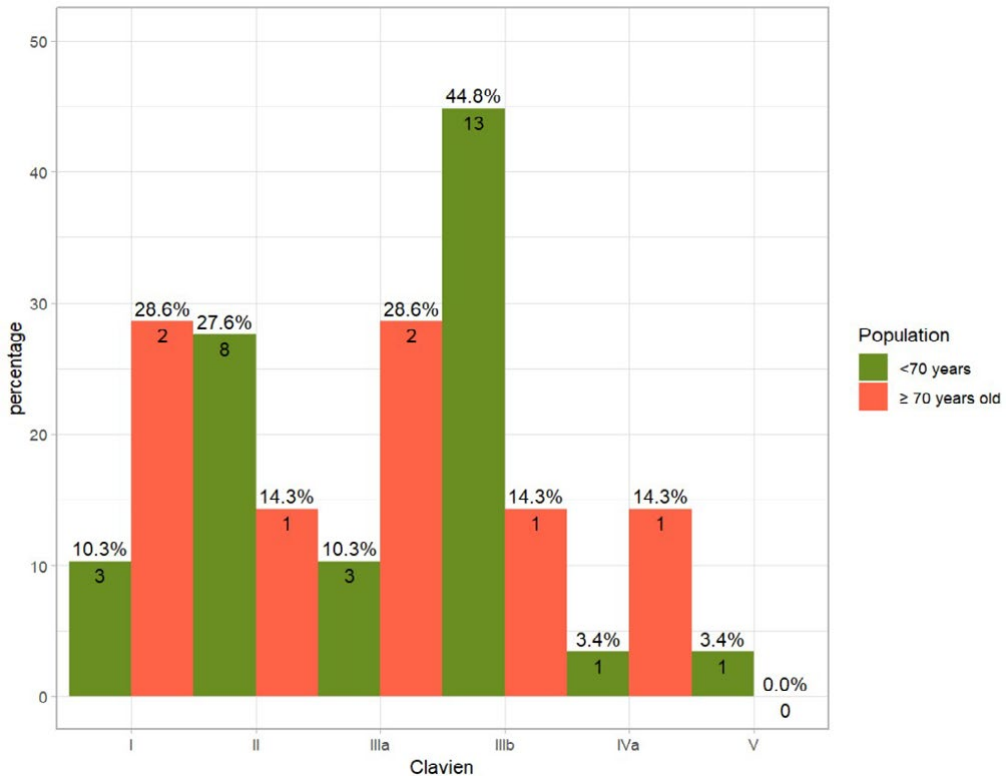
Distribution of complications in elderly and nonelderly patients according to the Clavien–Dindo classification system. [Color figure can be viewed at wileyonlinelibrary.com]

A total of 36 patients (31.5%) experienced 64 complications following TORS. Although there was a trend toward fewer complications in the elderly population, no statistically significant difference was observed in the rate of complications between the elderly (seven out of 37 patients, 18.9%) and nonelderly (29 out of 77 patients, 37.6%) groups (*p* = 0.07). Both TORS‐related and non‐TORS‐related complications showed no significant differences between the elderly and nonelderly populations, as indicated in Table 2. Of the 13 patients who experienced postoperative bleeding, 11 were from the nonelderly group (14.2%) and two from the elderly group (5.2%) (*p* = 0.16) indicating a trend toward less bleeding in elderly patients. Instances of bleeding were classified according to the Mayo classification as nine intermediate, three minor, and one major (massive lingual artery bleeding on the 14th day postoperatively). No significant differences in bleeding severity were observed between the elderly and nonelderly groups (*p* = 1). The classification of complications according to the Clavien–Dindo classification is shown in Figure 2.

**TABLE 2:** Comparison of complications between the two groups.

	< 70 years old *n* = 77 (%)	≥ 70 years old *n* = 37 (%)	*p*
Complications			0.072^a^
Yes	29 (37.6)	7 (18.9)	
No	48 (62.4)	30 (81.1)	
Complications (TORS—non‐TORS)			0.225
TORS	12 (15.6)	4 (10.8)	
non‐TORS	13 (16.9)	3 (8.1)	
TORS and non‐TORS	4 (5.2)	—	
Complications (Clavien–Dindo)			0.151
Minor [1, 2]	11 (14.3)	3 (8.1)	
TORS	3 (3.9)	2 (5.4)	
non‐TORS	8 (10.4)	1 (2.7)	
TORS and non‐TORS	—	—	
Major [3‐4‐5]	18 (23.3)	4 (10.8)	
TORS	9 (11.7)	1 (2.7)	
non‐TORS	5 (6.5)	3 (8.1)	
TORS and non‐TORS	4 (5.2)	—	
Bleeding—Mayo classification			1
Minor	3 (3.9)	0	
Intermediate	7 (9.1)	2 (5.4)	
Major	1 (1.3)	0	

^a^ Close to statistical significance.

### 3.4 Survival Outcomes

**FIGURE 3:**
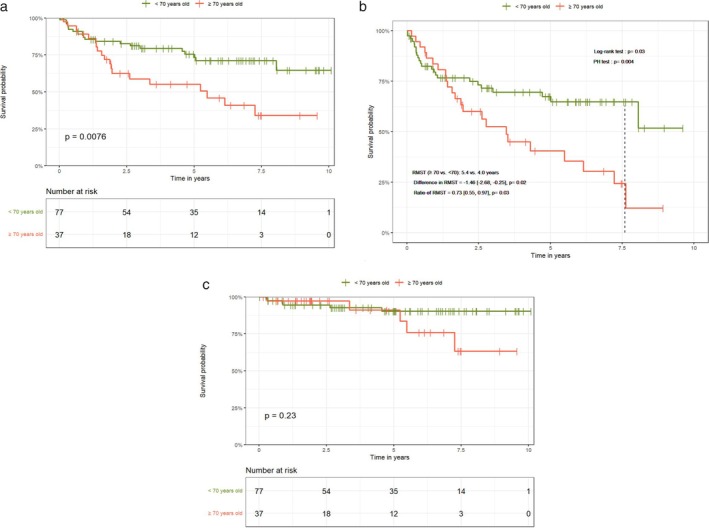
Survival plots of both groups showing (a) overall survival, (b) recurrence‐free survival, and (c) disease‐specific survival. [Color figure can be viewed at wileyonlinelibrary.com]

OS, RFS, and DSS rates at 12, 60, and 90 months for the entire cohort were as follows: OS 85.8%, 67.4%, 53.6%; RFS 79.9%, 56.4%, 42.5%; and DSS 94%, 88.6%, 82.3%, respectively. Elderly patients had worse OS and RFS rates than nonelderly counterparts; however there was no difference in DSS rates between the groups (Figure 3a–c). OS and RFS rates at 12, 60, and 90 months according to groups were as follows: OS 86.2%, 50.5%, 34.0% for the elderly group versus OS 84.2%, 73.3%, 64.6% for the nonelderly; and RFS 80.7%, 35.4%, 12.1% for the elderly group versus 78%, 64.7%, 51.8% for the nonelderly, respectively.

Univariate and multivariate analyses for OS across both groups are presented in Tables 3 and 4. For the elderly group, having a tumor located at the larynx vs. oropharynx and past radiotherapy were associated with increased HRs for OS in the multivariate analysis (HR 9.84 [95% CI: 2.55–37.97], *p* < 0.001 and HR 15.84 [95% CI: 3.88–64.60], p < 0.001, respectively). In the nonelderly group, lymphovascular invasion and extracapsular spread were associated with increased HRs for OS in the multivariate analysis (HR 12.94 [95% CI: 2.93–57.19], *p* < 0.001, HR 4.78 [95% CI: 1.07–21.28], *p* = 0.04, respectively).

**TABLE 3:** Multivariate analysis for overall survival in nonelderly patients.

	Univariate analysis		Multivariate analysis	
	*p*	HR (95% CI)	*p*	HR (95% CI)
Gender				
Female vs. male	0.659	1.24 [0.48–3.23]		
Stage				
Advanced (III‐IV) vs. early (0‐I‐II)	0.994	1.00 [0.40–2.52]		
cT classification				
cT2 vs. cT1	0.479	1.47 [0.51–4.24]		
cT3‐4 vs. cT1	**0.041**	10.00 [1.10–91.22]		
cTis/x vs. cT1	0.173	2.23 [0.70–7.04]		
cN classification				
cN1 vs. cN0	0.173	0.36 [0.08–1.58]		
cN2‐3 vs. cN0	0.862	0.91 [0.32–2.56]		
p16 status				
Negative vs. positive	**0.029**	0.25 [0.07–0.87]		
Localization				
Larynx vs. oropharynx	0.998	—		
Unknown primary vs. oropharynx	0.858	0.83 [0.11–6.32]		
Oral cavity vs. oropharynx	0.802	0.83 [0.19–3.63]		
Other vs. oropharynx	0.312	2.85 [0.37–21.67]		
Past primary				
Yes vs. no	**0.005**	3.55 [1.45–8.73]		
Past radiation				
Yes vs. no	**< 0.001**	6.61 [2.72–16.06]		
Synchronous tumor				
Yes vs. no	0.402	1.69 [0.49–5.80]		
Feeding tube insertion				
Yes vs. no	0.748	0.84 [0.28–2.51]		
Neck dissection				
Yes vs. no	**0.020**	0.35 [0.14–0.85]		
Tracheotomy				
Yes vs. no	0.998	—		
Frozen section				
Negative vs. none	0.130	0.37 [0.10–1.35]		
Positive vs. none	**0.023**	5.27 [1.27–25.93]		
Length of stay				
2 weeks vs. 1 week	0.696	1.29 [0.36–4.60]		
3 weeks vs. 1 week	0.860	0.86 [0.16–4.71]		
More than 3 weeks vs. 1 week	**0.007**	5.49 [1.58–19.02]		
Complications				
Yes vs. no	0.084	2.18 [0.90–5.27]		
pT classification				
pT2 vs. pT1	0.595	1.29 [0.50–3.36]		
pT3‐4 vs. pT1	0.208	3.82 [0.47–30.90]		
pTis/x vs. pT1	0.790	0.81 [0.17–3.78]		
Postoperative radiotherapy				
Yes vs. no	0.122	0.45 [0.16–1.24]		
Revision for margins				
Yes vs. no	0.730	1.18 [0.45–3.09]		
Initial margin status				
[1–3] mm vs. > 3 mm	0.310	1.72 [0.60–4.92]		
1 mm vs. > 3 mm	0.161	2.19 [0.73–6.56]		
Lymphovascular invasion				
Yes vs. no	**< 0.001**	7.55 [2.97–19.17]	**< 0.001**	12.94 [2.93–57.19]
Perineural invasion				
Yes vs. no	0.187	2.29 [0.67–7.84]		
Extracapsular spread				
Yes vs. no	**0.032**	4.57 [1.14–18.32]	**0.040**	4.78 [1.07–21.28]
CCI				
Moderate vs. mild	0.729	1.43 [0.70–10.98]		
Severe vs. mild	0.460	222 [0.27–18.48]		

*Note*: Bold indicates statistical significance.

**TABLE 4:** Multivariate analysis for overall survival in elderly patients.

	Univariate analysis		Multivariate analysis	
	*p*	HR (95% CI)	*p*	HR (95% CI)
Gender				
Female vs. male	0.126	0.42 [0.14–1.28]		
Stage				
Advanced (III‐IV) vs. early (0‐I‐II)	0.827	0.89 [0.32–2.49]		
cT classification				
cT2 vs. cT1	0.410	0.64 [0.22–1.85]		
cT3‐4 vs. cT1	0.481	2.15 [0.26–18.01]		
cTis/x vs. cT1	0.992	1.01 [0.30–3.36]		
cN classification				
cN1 vs. cN0	0.121	0.30 [0.07–1.37]		
cN2‐3 vs. cN0	0.843	1.11 [0.39–3.20]		
p16 status				
Negative vs. positive	0.116	0.44 [0.16–1.23]		
Localization				
Larynx vs. oropharynx	**0.009**	2.43 [0.83–7.11]	**< 0.001**	9.84 [2.55–37.97]
Unknown primary vs. oropharynx	0.973	1.03 [0.22–4.85]		
Oral cavity vs. oropharynx	0.537	1.94 [0.24–15.92]		
Other vs. oropharynx	0.504	1.70 [0.36–8.05]		
Past primary				
Yes vs. no	**0.001**	5.79 [2.03–16.53]		
Past radiation				
Yes vs. no	**0.004**	4.43 [1.63–12.04]	**< 0.001**	15.84 [3.88–64.60]
Synchronous tumor				
Yes vs. no	0.509	1.45 [0.48–4.38]		
Feeding tube insertion				
Yes vs. no	0.252	0.57 [0.22–1.48]		
Neck dissection				
Yes vs. no	0.261	0.59 [0.23–1.49]		
Tracheotomy				
Yes vs. no	—	—		
Frozen section				
Negative vs. none	0.09	0.33 [0.09–1.18]		
Positive vs. none	—	—		
Length of stay				
2 weeks vs. 1 week	0.279	0.47 [0.12–1.83]		
3 weeks vs. 1 week	0.169	0.33 [0.07–1.60]		
More than 3 weeks vs. 1 week	0.389	1.60 [0.55–4.69]		
Complications				
Yes vs. no	0.994	1.01 [0.29–3.48]		
pT classification				
pT2 vs. pT1	0.895	1.08 [0.37–3.17]		
pT3‐4 vs. pT1	0.998	—		
pTis/x vs. pT1	0.860	1.11 [0.35–3.57]		
Postoperative radiotherapy				
Yes vs. no	0.839	0.90 [0.32–2.53]		
Revision for margins				
Yes vs. no	0.640	1.25 [0.49–3.18]		
Initial margin status				
[1–3] mm vs. > 3 mm	0.300	1.96 [0.55–6.96]		
1 mm vs. > 3 mm	0.251	2.41 [0.54–10.78]		
Lymphovascular invasion				
Yes vs. no	0.908	0.89 [0.12–6.70]		
Perineural invasion				
Yes vs. no	0.116	2.18 [0.83–5.74]		
Extracapsular spread				
Yes vs. no	0.626	0.72 [0.19–2.73]		
CCI				
Severe vs. moderate	0.998	∞ [0‐∞]		

*Note*: Bold indicates statistical significance.

Regarding RFS, univariate and multivariate analyses for both groups are presented in Tables 5 and 6. For the elderly group, having a past primary tumor and lymphovascular invasion were associated with increased HRs for RFS in the multivariate analysis (HR 6.85 [95% CI: 2.44–19.24], *p* < 0.001 and HR 16.23 [95% CI: 2.85–92.45], *p* < 0.01, respectively). In the nonelderly group having a past primary tumor and lymphovascular invasion were associated with increased HRs for RFS in the multivariate analysis (HR 3.75 [95% CI: 1.61–8.73], *p* < 0.01 and HR 5.05 [95% CI: 2.03–12.55], *p* < 0.001, respectively).

**TABLE 5:** Multivariate analysis for recurrence‐free survival in nonelderly patients.

	Univariate analysis		Multivariate analysis	
	*p*	HR (95% CI)	*p*	HR (95% CI)
Gender				
Female vs. male	0.741	1.16 [0.48–2.81]		
Stage				
Advanced (III‐IV) vs. early (0‐I‐II)	0.965	0.98 [0.42–2.31]		
cT classification				
cT2 vs. cT1	0.256	1.72 [0.68–4.37]		
cT3‐4 vs. cT1	0.158	4.57 [0.56–37.63]		
cTis/x vs. cT1	0.451	1.52 [0.51–4.54]		
cN classification				
cN1 vs. cN0	0.091	0.28 [0.06–1.23]		
cN2‐3 vs. cN0	0.693	1.20 [0.49–2.94]		
p16 status				
Negative vs. positive	**0.036**	0.31 [0.11–0.93]		
Localization				
Larynx vs. oropharynx	0.998	—		
Unknown primary vs. oropharynx	0.729	0.70 [0.09–5.25]		
Oral cavity vs. oropharynx	0.475	0.59 [0.14–2.53]		
Other vs. oropharynx	0.255	3.24 [0.43–24.57]		
Past primary				
Yes vs. no	**< 0.001**	4.20 [1.83–9.63]	**< 0.01**	3.75 [1.61–8.73]
Past radiation				
Yes vs. no	**< 0.001**	4.51 [1.94–10.50]		
Synchronous tumor				
Yes vs. no	0.638	1.34 [0.40–4.51]		
Feeding tube insertion				
Yes vs. no	0.634	0.786 [0.29–2.12]		
Neck dissection				
Yes vs. no	**0.016**	0.37 [0.16–0.83]		
Tracheotomy				
Yes vs. no	0.998	—		
Frozen section				
Negative vs. none	0.082	0.27 [0.06–1.18]		
Positive vs. none	0.097	3.47 [0.80–15.09]		
Length of stay				
2 weeks vs. 1 week	0.796	0.87 [0.29–2.58]		
3 weeks vs. 1 week	0.649	0.72 [0.18–2.91]		
More than 3 weeks vs. 1 week	0.072	2.76 [0.92–8.32]		
Complications				
Yes vs. no	0.248	1.61 [0.72–3.62]		
pT classification				
pT2 vs. pT1	0.503	1.34 [0.57–3.16]		
pT3‐4 vs. pT1	0.425	2.31 [0.30–18.07]		
pTis/x vs. pT1	0.570	0.64 [0.14–2.93]		
Postoperative radiotherapy				
Yes vs. no	0.279	0.61 [0.25–1.49]		
Revision for margins				
Yes vs. no	0.844	1.009 [0.45–2.64]		
Initial margin status				
[1–3] mm vs. > 3 mm	0.570	1.32 [0.50–3.48]		
1 mm vs. > 3 mm	0.215	1.85 [0.70–4.90]		
Lymphovascular invasion				
Yes vs. no	**< 0.001**	5.98 [2.43–14.74]	**< 0.001**	5.05 [2.03–12.55]
Perineural invasion				
Yes vs. no	0.192	2.25 [0.66–7.65]		
Extracapsular spread				
Yes vs. no	**0.01**	5.15 [1.48–17.91]		
CCI				
Moderate vs. mild	0.432	0.55 [0.12–2.44]		
Severe vs. mild	0.944	1.06 [0.22–5.00]		

*Note*: Bold indicates statistical significance.

**TABLE 6:** Multivariate analysis for recurrence‐free survival in elderly patients.

	Univariate analysis		Multivariate analysis	
	*p*	HR (95% CI)	*p*	HR (95% CI)
Gender				
Female vs. male	0.288	0.61 [0.25–1.51]		
Stage				
Advanced (III‐IV) vs. early (0‐I‐II)	0.155	1.86 [0.79–4.35]		
cT classification				
cT2 vs. cT1	0.932	0.96 [0.36–2.57]		
cT3‐4 vs. cT1	0.461	2.23 [0.27–18.64]		
cTis/x vs. cT1	0.540	1.40 [0.47–4.16]		
cN classification				
cN1 vs. cN0	0.589	0.75 [0.26–2.14]		
cN2‐3 vs. cN0	0.883	0.92 [0.32–2.69]		
p16 status				
Negative vs. positive	0.256	0.59 [0.23–1.47]		
Localization				
Larynx vs. oropharynx	**< 0.01**	4.25 [1.49–12.07]		
Unknown primary vs. oropharynx	0.521	1.48 [0.45–4.92]		
Oral cavity vs. oropharynx	0.728	1.45 [0.18–11.77]		
Other vs. oropharynx	0.727	1.32 [0.28–6.17]		
Past primary				
Yes vs. no	**< 0.001**	4.92 [1.92–12.61]	**< 0.001**	6.85 [2.44–19.24]
Past radiation				
Yes vs. no	**< 0.01**	3.99 [1.52–10.47]		
Synchronous tumor				
Yes vs. no	0.847	1.11 [0.38–3.3]		
Feeding tube insertion				
Yes vs. no	0.101	0.48 [0.20–1.16]		
Neck dissection				
Yes vs. no	0.597	0.78 [0.32–1.93]		
Tracheotomy				
Yes vs. no	1	—		
Frozen section				
Negative vs. none	**0.037**	0.26 [0.07–0.92]		
Positive vs. none	0.839	0.81 [0.11–6.22]		
Length of stay				
2 weeks vs. 1 week	0.542	0.69 [0.21–2.27]		
3 weeks vs. 1 week	0.165	0.32 [0.06–1.60]		
More than 3 weeks vs. 1 week	**0.042**	3.07 [1.04–9.08]		
Complications				
Yes vs. no	0.292	1.81 [0.60–5.47]		
pT classification				
pT2 vs. pT1	0.801	1.15 [0.39–3.37]		
pT3‐4 vs. pT1	0.162	3.00 [0.64–14.00]		
pTis/x vs. pT1	0.677	1.26 [0.43–3.68]		
Postoperative radiotherapy				
Yes vs. no	0.533	1.38 [0.50–3.77]		
Revision for margins				
Yes vs. no	0.763	1.14 [0.48–2.73]		
Initial margin status				
[1–3] mm vs. > 3 mm	0.470	1.47 [0.63–7.73]		
1 mm vs. > 3 mm	0.218	2.20 [0.52–4.13]		
Lymphovascular invasion				
Yes vs. no	**0.024**	5.98 [1.26–28.40]	**< 0.01**	16.23 [2.85–92.45]
Perineural invasion				
Yes vs. no	**< 0.01**	4.10 [1.57–10.71]		
Extracapsular spread				
Yes vs. no	0.405	1.57 [0.54–4.56]		
CCI				
Severe vs. moderate	0.998	∞ [0‐∞]		

*Note*: Bold indicates statistical significance.

## 4 Discussion

The general aging of the population and the increase in the incidence of HPV‐driven oropharyngeal cancer are leading to an increase in the number of elderly patients who are expected to undergo TORS and associated adjuvant treatment modalities [8, 13, 14]. Balancing the risks of TORS with its potential benefits in elderly patients is of paramount importance in clinical decision‐making.

With regard to the patient and tumor characteristics, a significant difference between the two groups was observed in terms of tumor location. A higher incidence of larynx and unknown primary sites was observed in the elderly population, while a higher incidence of oropharynx and oral cavity was observed in the nonelderly patients. A further notable distinction was observed in the initial safe and close margin status, which we hypothesize can be attributed to the variation in tumor subsites. While it is technically feasible to include more tissue adjacent to the tumor in the oropharynx region, the supraglottic larynx may lack this possibility due to its more convoluted nature and the presence of less resectable neighboring tissue. This may be a reason why less wide margins were observed in the elderly group. Furthermore, a reduced tendency for postoperative chemotherapy was observed among the elderly population, although it was close to reaching statistical significance. This outcome is most likely attributable to findings from the meta‐analysis of chemotherapy in head and neck cancer (MACH‐NC), which demonstrated a decrease in the efficacy of adjuvant chemotherapy in patients over 70, influencing our tumor board's decisions [15]. In a cohort including elderly patients with HPV‐positive oropharynx tumor by Parhar et al., the rate of adjuvant chemoradiotherapy was 26% [8]. Similarly to our study, Philips et al. reported a reduced incidence of adjuvant chemoradiation in elderly patients compared to nonelderlies; however, in contrast to our study, the incidence of adjuvant radiotherapy was higher in elderlies [6]. The variations in the institutions' multidisciplinary tumor boards', tendency for the selection of adjuvant treatment in the elderly patients, or patient‐related factors, could have contributed to this discrepancy. Nevertheless, a recent paper including 10 430 patients from the NCDB who had undergone TORS for oropharynx cancer (OPC) reported that elderly patients were less frequently administered adjuvant radiotherapy and/or chemotherapy, comparable to the results obtained in this study [9].

The prevalence of tracheotomy, feeding tube and gastrostomy status has been identified as the most commonly reported functional outcome measures following TORS [16]. The results of the present study indicate no difference between elderly and nonelderly patients in any of these parameters, which is in line with the findings of previous literature [3]. Recent systematic reviews on functional outcomes following TORS reported a chronic gastrostomy tube dependence rate between 0% and 7%, which was also comparable to the rate observed in the present study [16, 17]. The results of the present study also did not show any difference in chronic gastrostomy tube dependency between the compared groups. In a study conducted by Dziegielewski et al. patients over the age of 55 exhibited a nearly fivefold increased probability of requiring gastrostomy tubes following TORS in comparison with younger patients [18]. In the present study, we did not observe any variation in feeding tube rates between elderly and younger patients after TORS. This may be attributed to differing age cutoffs across studies, which could introduce a confounding effect, making direct comparisons less reliable. Moreover, the tendency to apply more aggressive swallowing rehabilitation in elderly patients may have also contributed to this lack of significance. With regard to the assessment of swallowing function, some studies utilized FOSS scores in a manner consistent with our own study, while others employed different tools, including the M.D. Anderson Dysphagia Inventory composite score (MDADI), videofluoroscopy and the dynamic imaging grade of swallowing toxicity (DIGEST), or the functional oral intake scale (FOIS) [19, 20, 21, 22, 23]. The findings of this study suggest that TORS does not induce any substantial alterations in swallowing function in the elderly population, despite their elevated FOSS scores at all three time periods compared to the nonelderly group. Nonetheless, the swallowing function of the nonelderly population appears to be considerably impacted by surgery, yet it recovers to its preoperative baseline at the final follow‐up. In their series of patients who underwent TORS for locally advanced oropharyngeal cancer, Gross et al. also reported that patients with poor (FOSS 3–5) swallowing outcomes were older (mean age: 62.5 years) compared to those with acceptable (FOSS 0–2) swallowing function (mean age: 58 years) (OR 1.06, 95% CI 1.00–1.12) [24]. Philips et al. reported comparable swallowing recovery between elderly and nonelderly patients in their predominantly HPV (+) OPC cohort at 3 months, 1 and 2 years postoperatively, as indicated by FOIS scores and the need for enteral feeding [6]. Although our data were obtained from a markedly different patient population, with only 33% (nonelderly) to 37% (elderly) p16‐positive patients and 32% of elderly patients treated for secondary primaries, we still confirmed good functional outcomes in the elderly group. Overall, the results of this study support that TORS can be safely performed in elderly populations with respect to functional outcomes. We also believe that prehabilitation with intensified swallowing exercises may be particularly beneficial for patients who already have poor swallowing function at baseline.

Another concern for elderly candidates of TORS is the existence of perioperative surgical risks. The identification of these risks is of paramount importance for two fundamental reasons. First, it ensures that patients receive comprehensive information about the potential consequences of the procedure. Second, it facilitates the optimal decision‐making process between surgical and nonsurgical treatment options. It is important to note that as individuals age, they tend to become more frail, with 10% of adults aged 50–64 and 43.7% of those aged ≥ 65 affected by this phenomenon [25]. In a study investigating post‐TORS complications, Hay et al. reported a 47% complication rate which was higher than our cohort [12]. In their systematic review investigating complications following salvage TORS, Turner et al. reported an overall complication rate of 33.6% [26]. Consistent with our findings, Philips et al. observed no significant difference in the rates of bleeding between elderly and nonelderly patients [3]. Additionally, our study classified the severity of bleeding, which provides further detail on this complication in both age groups and contributes to a better understanding of the overall complication profile. In a study by Hay et al. that included 122 TORS cases, patients aged over 60 years were reported to have higher rates of both major complications (grade 3‐4‐5) and overall complication rates. Furthermore, they found that being older than 60 years old increased the odds of overall complications by 2.7 fold [12]. In contrast, our data showed a trend toward older individuals experiencing fewer complications, which may seem unexpected. We believe this may reflect selection bias, as elderly patients were selected with greater caution for surgery. Furthermore, refusal of surgery may have been more common in the elderly group. However, the inconsistencies in the age cut‐off between the studies render it difficult to make a confident comparison between the outcomes.

The OS and RFS rates of the nonelderly group were found to be better than those of the elderly patients. However, the DSS rates were similar; other risk factors, rather than the disease itself, were likely the underlying cause of the observed differences between the groups. Phillips et al. reported an OS HR of 4.93 for patients with prior treatment with chemoradiation in their total cohort, which included both elderly and nonelderly patients [27]. A separate regression analysis for both groups in our study allowed us to identify that a history of radiation therapy was associated with an increased hazard ratio for OS in elderly patients, but not in nonelderly patients. The proportion of patients with a prior primary tumor in our entire cohort was comparable to that reported by Hay et al. (28.7%) [12]. Nonelderly patients exhibited different risk factors for OS, such as lymphovascular invasion and extracapsular spread, compared to elderly patients. This difference may be attributed to the lower prevalence of competing risks in nonelderly populations, allowing other factors to have a greater impact on the regression model. Notably, RFS analyses identified the same risk factors in both the elderly and nonelderly groups. Moreover, despite higher CCI scores in elderly patients, CCI was not identified as a risk factor for survival in either elderly or nonelderly groups, suggesting that survival in the elderly population also depends largely on treatment‐related factors.

This is the first study presenting a comparison of survival, morbidity, and functional outcomes of elderly versus nonelderly TORS patients in a population characterized by a low p16‐positive and OPC rate, but high secondary primary rate. Multivariate analyses for survival predictors stratified by age revealed different risk factors, thereby increasing the granularity of the results, which, to the best of our knowledge, have not been previously reported.

This study is not without limitations. First, functional outcomes were evaluated only by the FOSS score, which does not represent patient‐reported swallowing function. The FOSS score was chosen primarily because of its feasibility in a retrospective design. Furthermore, speech parameters, which are also an important component of functionality, were not assessed in this study. Finally, the definition of “elderly” patients varies in the literature. The age cut‐off in this study was 70 years, as it was used in many previous papers [3, 8, 9]. However, other papers use different age thresholds [12, 18]. This may have altered the results of the statistical analyses.

## 5 Conclusion

Elderly patients had complication rates comparable to nonelderly patients following TORS. While TORS temporarily worsened swallowing in nonelderly patients within 3 months postoperatively, both groups returned to baseline at the last follow‐up, indicating no lasting impact. The nonelderly group showed better overall and RFS, while DSS remained similar. These findings support the safe use of TORS in elderly patients even in populations with a relatively low p16‐positive and OPC rate, without added morbidity and with comparable oncological outcomes.

## Conflicts of Interest

The authors declare no conflicts of interest.

## Supporting information


**Figure S1:**Distribution of FOSS scores for elderly and nonelderly patients at each time period.

## Data Availability

The data that support the findings of this study are available on request from the corresponding author. The data are not publicly available due to privacy or ethical restrictions.
