# Supplementary figures and images for: Transoral Robotic Surgery (TORS) for Head and Neck Cancer in the Elderly Population: Functional Outcomes, Survival, and Complications
Source: Head Neck. 2025 Nov 19;48(4):1016–30. doi: 10.1002/hed.70097 (PMC12972643; doi:10.1002/hed.70097)

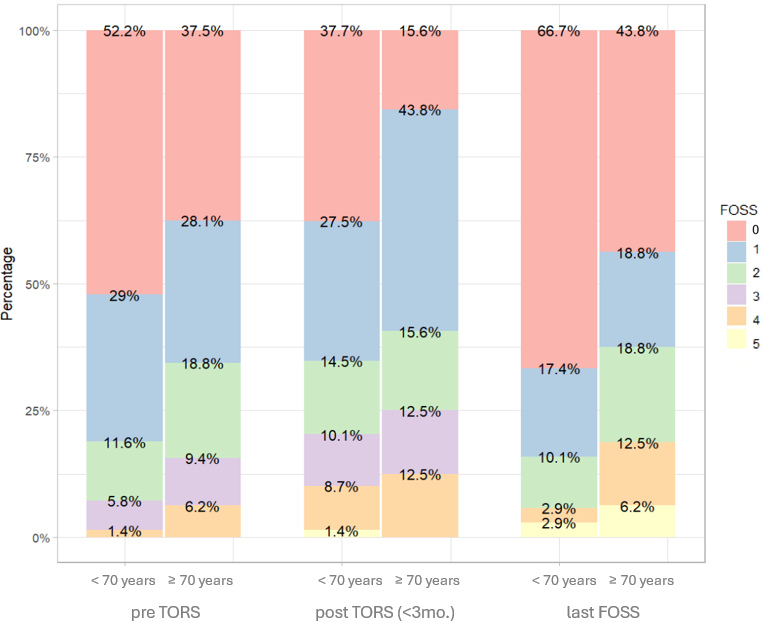

Supplement: Supplementary file 1 — Figure S1:Distribution of FOSS scores for elderly and nonelderly patients at each time period. [file HED-48-1016-s001.png]
